# Multi-modality trajectory prediction with the dynamic spatial interaction among vehicles under connected vehicle environment

**DOI:** 10.1038/s41598-024-53315-6

**Published:** 2024-02-04

**Authors:** Lisheng Jin, Xingchen Liu, Yinlin Wang, Zhuotong Han, Baicang Guo, Guofeng Luo, Xinliang Xu

**Affiliations:** Yanshan University, Qinhuangdao, 066000 China

**Keywords:** Mechanical engineering, Electrical and electronic engineering

## Abstract

Compared to non-connected vehicle environments, the connected vehicle environment establishes vehicle interconnection through communication technologies, resulting in more complex interaction, network topologies, and large-scale inputs. This complexity renders traditional trajectory prediction models, which rely primarily on inputting historical information of the target vehicle, inadequate for handling the complex and dynamic interactive lane-changing scenarios in connected vehicle environments. In a connected vehicle environment, it is necessary to propose a more targeted and stable lane-changing behavior prediction method based on vehicle traveling characteristics. Taking into account dynamic spatial interaction among vehicles, this study proposes a multi-modality trajectory prediction model called STA-LSTM to perform analysis on the potential interactive behaviors among vehicles under connected vehicle lane-changing scenarios, and specifically to expand the multi-modality feature input of the vehicle trajectory prediction model. The spatial grid occupancy method is used to model the interactions between vehicles. A space-dimensional attention mechanism is introduced to adaptively match the influencing weights of the surrounding vehicles with the target vehicle and to improve the interactive information extraction method. In addition, the attention module is incorporated into the LSTM decoder from the time dimension so that the established model can identify significant historical hidden features during each trajectory decoding process. To account for the uncertainty of trajectory prediction, the vectors of vehicle interactions are incorporated into contextual information to improve the reliability of prediction results and the robustness of the established model. Compared with conventional baseline models, the proposed model exhibited lower root mean square error (RMSE) and negative log-likelihood (NLL) values, and the RMSE values at different prediction times of 1s, 2s, 3s, 4s, and 5s are 0.46m, 1.15m, 1.89m, 2.84m, and 4.05m, respectively. This indicates that the proposed model can accurately predict the interactions between vehicles and the travel paths of surrounding target vehicles.

## Introduction

With the rapid progress of the automotive industry and rapid advancements in information transmission, software, and information technology services, the research community has focused on designing intelligent networking and a new industrial ecosystem^1,2^. Due to highly efficient vehicle-vehicle and vehicle-infrastructure information coordination in a connected vehicle environment, vehicles are able to transmit their operational information and receive traffic information from the surrounding environment. This enables the driver to avoid impending hazardous road conditions and invisible road hazards, thereby enhancing driving safety and the operational efficacy of road traffic^3,4^.

In the realm of connected vehicle ecosystems, drivers are confronted with the formidable task of assimilating and processing a labyrinth of intricate information emanating from an array of sources, including other vehicular entities, traffic management systems, and advanced intelligent traffic apparatuses. This encompasses a spectrum of real-time data streams, detailing the kinematic aspects of proximate road users, prevailing traffic conditions, systemic intercommunications, and pertinent cautionary advisories. The inherently composite and multifarious nature of this informational milieu exacerbates the cognitive processing burden and escalates the operational workload for drivers. Consequently, this leads to a detrimental impact on crucial aspects of driving such as the allocation of attentional resources, the accuracy of environmental perception, and the agility of vehicular maneuvering. In addressing these complexities, the advent of vehicle trajectory prediction technology within connected vehicle frameworks emerges as a pivotal aid. It equips drivers with enhanced tools for an incisive understanding and appraisal of the dynamic vicissitudes in traffic flow and vehicular behavior modalities of adjacent entities. This augmented cognizance significantly bolsters decision-making efficacy, thereby providing an indispensable scaffold for the assurance of vehicular operational safety. Specifically, under a connected vehicle environment, lane change incidents have become more complex and diverse, as have the interactions between vehicles^5^. Therefore, vehicle trajectory prediction under a connected vehicle environment has become an extremely challenging task^6^.

Depending on the modeling abstraction level, there are three varieties of vehicle trajectory prediction methods^7^: physics-based methods, pattern-based methods, and interactive perception-based methods. Using physics-based methods, short-term prediction of vehicle trajectory can be achieved based on the dynamic and kinematic rules governing the vehicle. Veerar et al.^8^ focused on city intersections and proposed a deterministic sampling-based Kalman filtering prediction algorithm. This algorithm was highly accurate and robust. Toledo et al.^9^ collected vehicle position information by using GPS and IMU sensors, and established an extended Kalman filter based on the interactive multiple model that can predict lane change of the vehicles on high-speed roads. The aforementioned methods were developed based on low-level vehicle motion characteristics, resulting in a large variance in long-term prediction. Therefore, these algorithms are limited to short-term vehicle trajectory prediction.

The pattern-based methods consist of two steps. First, the goal and motive of the target vehicle were established in order to build a high-level representation of the motion state. After that, the vehicle interactions can be utilized as prior knowledge to predict the vehicle’s trajectory. Using a support vector machine (SVM), Aoude et al.^10^ accurately identified the vehicle’s turning intention under crossroad scenarios and performed risk assessment on vehicle trajectory based on a rapidly-exploring random tree algorithm (random number algorithm), which serves as a benchmark for an assistant driving anti-collision system. Ji et al.^11^ focused on high-speed lane-changing scenarios and developed an intention recognition module and a trajectory prediction module using long- and short-term memory networks. Based on this network, the vehicle’s predicted trajectory was characterized by possibility distribution. Most of these methods only considered the vehicles’ past states for trajectory inference. However, the future driving trajectory of a traffic vehicle depends not only on its own previous states, but also on the type of vehicle. Different types of vehicles affect the motion patterns differently. In addition, the vehicles exhibit close and extensive interactions with their neighbors. The methods based on behavior motive do not thoroughly account for the spatial interactions between vehicles, thereby severely limiting the long-term prediction performance of trajectory prediction algorithms.

The trajectory prediction methods based on behavioral maneuvering learn the driving intent of vehicles from observed trajectory data and use this driving intent as prior knowledge when making predictions. Unlike methods based on physics, these methods can predict the long-term trajectory of vehicles. The trajectory prediction methods divide the trajectory prediction task into two steps. First, identify the intent and maneuvers of the target vehicle in order to generate a high-level expression of the motion state, such as lane changing and lane maintenance, and then output the predicted trajectory over time. Hermes et al.^12^ combined the RBF classifier and mean shift clustering algorithm to predict the long-term behavior of vehicles under turning conditions using data such as vehicle curvature and speed. Aoude et al.^10^ accurately identified the turning intention of vehicles at intersections using a support vector machine and a fast search random number algorithm (RRT) for conducting hazard assessment of vehicle trajectories, providing a benchmark for auxiliary driving anti-collision systems. Althoff et al.^13^ observed that most of the aforementioned maneuver-based prediction methods only infer the target vehicle’s trajectories by considering its dynamic information. However, the type of vehicle can also affect the movement mode. It is noteworthy that there is a close and deep-seated interaction between the vehicles and surrounding traffic participants. However, these methods do not take into account the spatiotemporal interactions between vehicles, limiting the long-term efficacy of trajectory prediction algorithms.

Taking into account the effect of the surrounding traffic environment on the target vehicle, interactive perception-based methods establish interaction-based models for predicting the trajectory of the target vehicle. In the past, researchers proposed a traditional model of social forces^14^ that accounted for both attraction and repulsion among pedestrians. However, these models were constructed artificially and lacked the flexibility to accurately reflect the complexity of the real-world traffic environment.

Due to a significant increase in GPU hash rate and data quality, data-driven methods based on deep learning have progressively become mainstream in interactive perception-based prediction. For example, LSTM^15^, GRU^16^, Bi LSTM^17^, and others are widely used in data-driven prediction models due to their powerful temporal modeling capabilities. Alahi et al.^18^ first proposed the concept of social LSTM in pedestrian trajectory prediction and encoded the pedestrian’s previous positions using a weight-shared LSTM network. Moreover, based on the relative positions of the pedestrians, the encoded vectors representing social interactions among traffic participants were added to the social pool. Hou et al.^19^ proposed a trajectory prediction method based on structural LSTM in which each LSTM unit is designated to an inter-active vehicle. This LSTM coding information may have a diametric relationship with the hidden state of the adjacent neighboring vehicles in order to share unit information of the interactive vehicle and hidden states. In order to characterize the observed vehicle interaction, Deo et al.^20^ improved the social pool in Social LSTM, extracted the temporal characteristics of vehicle trajectory by using LSTM units, and constructed a 13*3 spatial network to represent the spatial position interactions among vehicles. Moreover, the generated network was described using CNN in order to preserve the vehicle’s position relationship and obtain satisfactory prediction results. However, CS-LSTM ignores the influencing weight of other vehicles on the target vehicle, and performed poorly in terms of capturing the dynamic interactive information, thereby restricting the prediction precision. To address this issue, Cai et al.^21^ proposed the environment-attention network (EA-Net), which employs a novel parallel structure consisting of a graph attention network (GAT) and convolutional social pool with compressed extraction mechanism acting as the environmental feature extraction module embedded in the LSTM encoder/decoder, which can effectively improve the prediction accuracy. Tian et al.^22^ designed a deep interaction encoding decoding model based on the attention mechanism, which enhanced the model’s prediction accuracy by integrating a GRU module into the attention mechanism.

In addition to LSTM, graph convolutional neural (GCN) networks, such as GISNet^23^ and SAGAT^24^, have been widely used in trajectory prediction tasks due to their strong learning capability of the potential correlations between multiple spatial distribution nodes. However, these methods must construct a map of all scenario objectives, which is quite time-consuming. This also restricted the applicability to actual conditions.

By analyzing the above methods, scholars have performed valuable explorations in the behavioral prediction of intelligent and connected vehicles. Methods based on deep learning are progressively becoming a viable means of solving technological issues. Inspired by this issue, this study examines in depth the interactive information between vehicles and their behavioral characteristics in a connected vehicle environment. Moreover, it thoroughly investigates the prospective laws underlying the data in a network environment using the robust information processing capabilities of deep learning from a data-driven perspective. Furthermore, the Multi-Spatiotemporal Attention-LSTM (STA-LSTM) considering dynamic spatial interaction among vehicles is proposed, and the superiority of the model in vehicle trajectory prediction is validated through comparison with the baseline model.

## Framework of the prediction model

**Figure 1:**
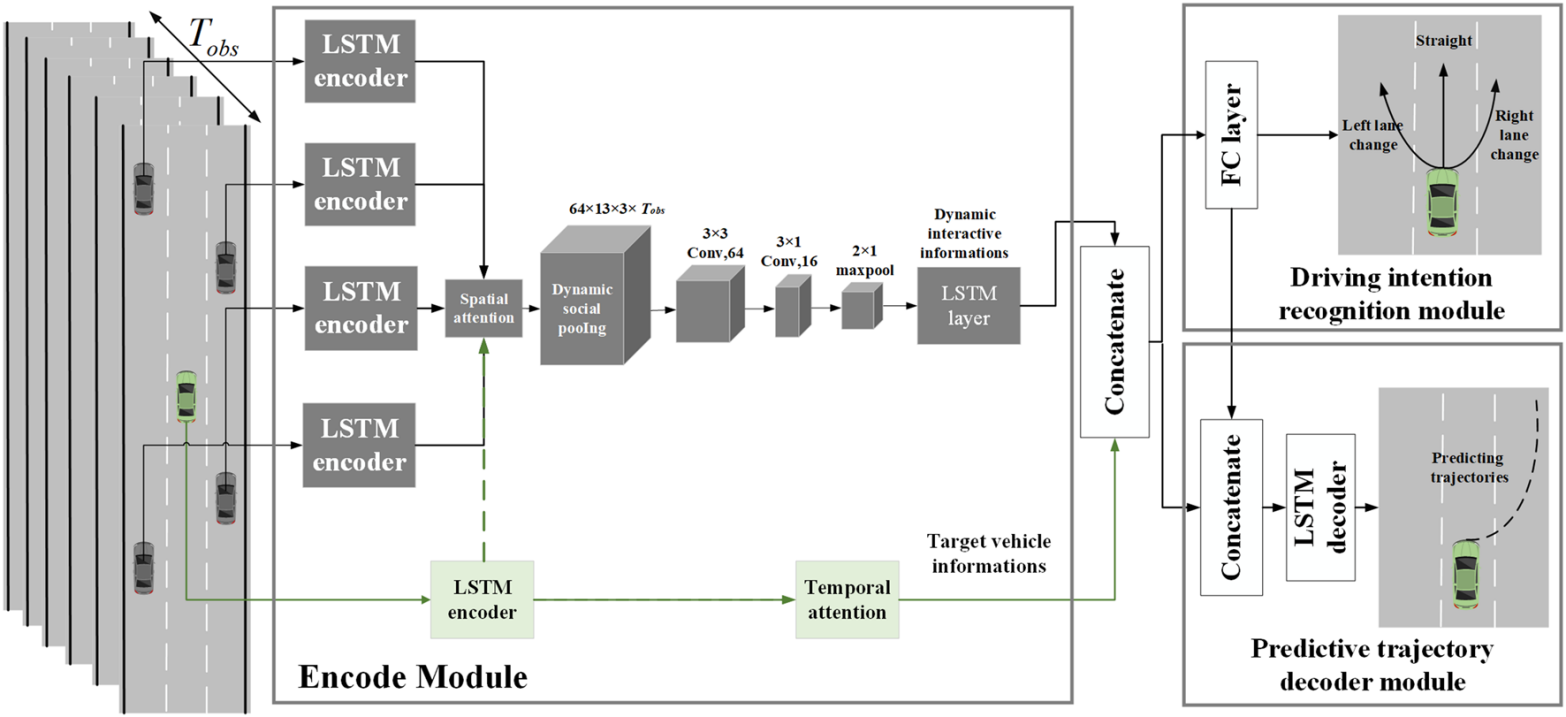
The established multi-modality trajectory prediction model based on the time-space attention model.

The overall framework of the proposed prediction model is shown in Fig. 1. First, the potential interactive behaviors among vehicles under connected vehicle lane change scenarios are analyzed in depth. Then, the interactive relation among vehicles is modeled with the occupancy of spatial grids. Second, during the encoding-decoding process of the historical information of target vehicles, the temporal attention mechanism is implemented to solve single intermediate semantic vectors and information loss within the encoder-decoder framework. Afterward, during the modeling process of interactive information of surrounding vehicles, the encoded information of the surrounding vehicles is extracted for weight calculation by employing the core concept of reallocation of characteristic weights based on the attention mechanism and introducing the spatial attention mechanism. To achieve feature enhancement of interactive tensors, the features of strongly correlated interactive vehicles should be amplified while the features of weakly correlated interactive vehicles should be weakened. To fully preserve the spatial position information of interactive vehicles, the convolution pooling layer in the CS-LSTM prediction model is used to extract the interactive features between the target vehicle and the surrounding vehicles. Finally, the extracted features of the target vehicle and the interactive features are spliced. The possibility of vehicles interactions among the target vehicles is calculated by using the Softmax function. By feeding the interactions and interactive features of vehicles into the LSTM decoder, the final probability distribution of predicted vehicle trajectories is obtained.

Deo et al.^20^ proposed a convolutional social LSTM prediction model that can extract local information from interactive tensors using the space invariance of convolutional neural networks (CNN). Despite a precise representation of the relationship between the target and surrounding vehicles, the varying degrees of influence among the interactive vehicles are not taken into account. In real-world scenarios, the real-time behavior decision of the target vehicle is influenced by the surrounding vehicles, as indicated by the dynamic and interactive spatial correlation. In order to accurately obtain the influencing relationship among vehicles, the influencing weights of the surrounding vehicles can be adaptively matched to the target vehicle in space, based on the essential thought of the attention mechanism during the encoding phase. By extracting the spatial weight information of the interactive vehicles, the interactive features in social tensors are recalculated in order to overcome the limitation, i.e., the fixed acquisition of vehicle interactive features, by employing the CNN in the convolutional social LSTM model. The degree of importance of information at different moments is dynamically allocated in the time dimension so that the model can recognize historically significant information while avoiding information loss during encoding. Similarly, historical information can be fully utilized. The detailed implementation process is described below.

### Feature extraction network

In the parameter-sharing LSTM encoder, the target’s and surrounding vehicles’ historical features are inputted. The LSTM network, which consists primarily of the embedding layer and an LSTM-based encoding layer, is used to extract long- and short-term motion and interactive features.

The word embedding layer is designed as a fully-connected layer including 32 output nodes. The number of input nodes corresponds to the vehicle’s defining dimension. ReLU is used as the activation function. For the vehicle’s characteristics information $X_{t}$ in traffic scenario, a high-dimensional vector can be obtained by using the information embedding:

1 $$\begin{aligned} e_{t} =FC(X_{t};W_{e} ), \end{aligned}$$

where $W_{e}$ represents the connected weight of the word embedding layer and $X_{t}$ denotes the vehicle’s eigenvector at moment *t*.

The vehicle’s high-dimensional feature output from the word embedding layer is used as the input of the LSTM encoder. For achieving the balance between precision and efficiency of feature extraction network, the number of the hidden units in LSTM is set to 64. After coding, a 64-dimensional hidden state $h_{t}$ is obtained:

2 $$\begin{aligned} h_{t}=LSTM(h_{t-1},e_{t};W_{t} ), \end{aligned}$$

where $h_{t-1}$ represents the hidden vector of the vehicle feature at previous moment, and $W_{t}$ denotes the connected weight of the LSTM encoder. For complete historical time domain $T_{obs}$ , the characteristic tensor *H* consisting of historical hidden states obtained using the feature extraction network, with a dimension of $T_{obs\times 64}$ , can be written as:

3 $$\begin{aligned} H=(h_{1},h_{2},\cdots ,h_{T_{obs} } ). \end{aligned}$$

### Modeling of interactive information

**Figure 2:**
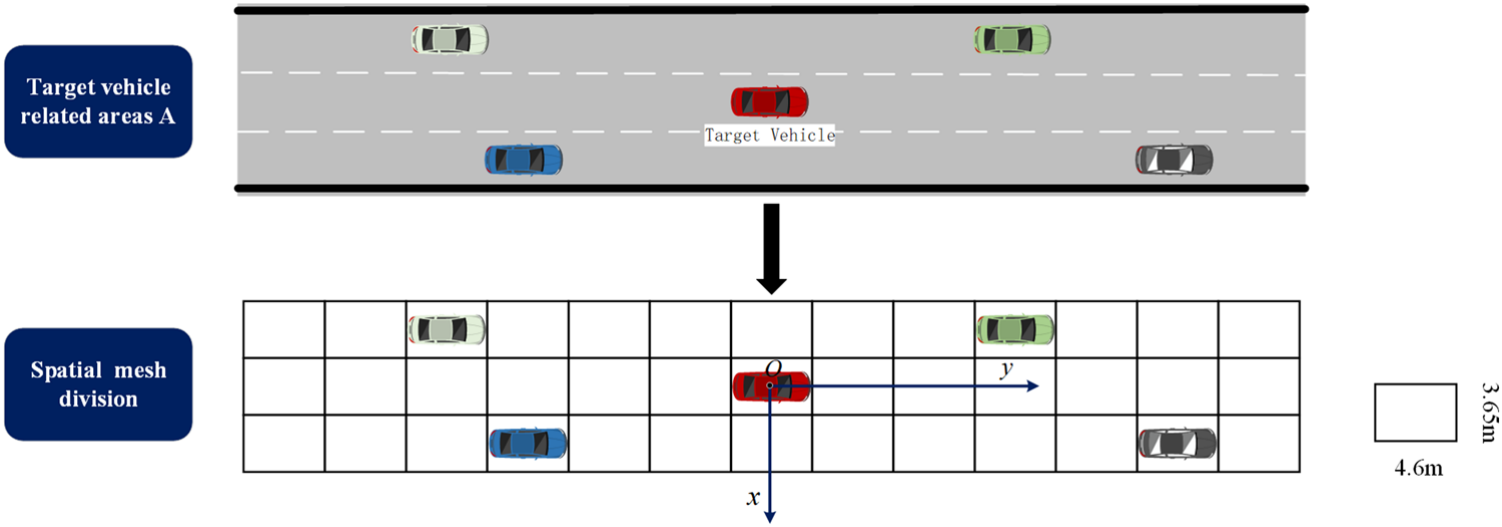
The spatial mesh generation and the establishment of the reference coordinate system.

The establishment of interactive models has always been a research focus in trajectory prediction studies. Similar to the CS-LSTM model, the interactive tensor of the vehicle is derived from the road structure where the vehicles are located. The neighboring space centered at the target vehicle can be divided into the spatial network of size 13*3. The network row represents the land over which the vehicle traveled, and the height of the row corresponds to the width of the lane. The distance between the columns in the network is 4.6 m, which represents the length of the vehicle. The established spatial network and the reference coordinate system are depicted in Fig. 2.

The following coordinate system is established for the predicted scenario to improve the performance of generalization during the modeling of interactive information. The origin of the coordinate o is fixed at the centroid of the target vehicle at time *t*, with the y-axis pointing in the target vehicle’s motion direction and the x-axis pointing perpendicular to the high-speed road.

The social characteristic tensors of the vehicles are obtained by using the feature extraction network. Through position embedding, these vectors are populated with the corresponding position of the interactive tensor. Based on the transverse and longitudinal position deviations of the surrounding vehicles relative to the target vehicle at time *t*, the positions of the neighboring vehicles’ hidden vectors in the interactive tensor are determined, denoted as (*m*, *n*) , can be written as:

4 $$\begin{aligned} \left\{ \begin{matrix}h_{nbrs}^{t}=\delta _{13\times 3}(h_{1}^{t},h_{2}^{t},\cdots ,h_{n}^{t} ) \\ m=floor(x_{i}^{t}-x_{hist}^{t}/l ) \\ n=floor(y_{i}^{t}-y_{hist}^{t}/d ) \end{matrix}\right. \end{aligned}$$

where $$\delta _{13\times 3}= \left\{ \begin{matrix}1,Vehicle\,within\,mesh \\ 0,Vehicle\,outside\,mesh\end{matrix}\right.$$ (1 represents the vehicle located in the grid and 0 represents the vehicle located in the grid), *m* and *n* represent the row number and the column number of the traffic participant in the spatial grid, respectively ($m\in (-1,0,1)$ and $n\in (-6,-5,\dots ,5,6)$). $floor(\cdot )$ represents the round-down function, and *l* and *d* denote the lane width and lane length, respectively.

**Figure 3:**
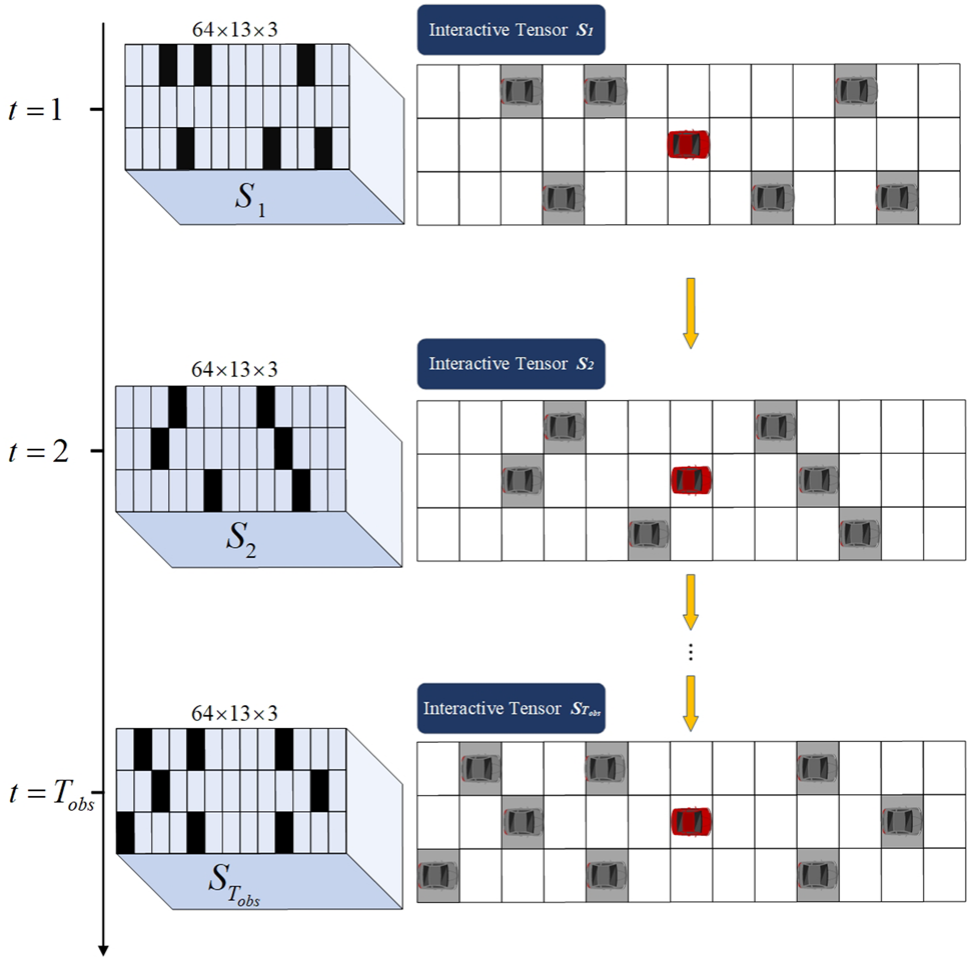
The dynamic interactive tensor.

According to Eq. (4), the hidden state $h_{t}^{1}, h_{t}^{2}, \dots ,h_{t}^{n}$ of the interactive information associated with the surrounding vehicle at moment *t* is filled by the corresponding position in the space grid, and the position of the unoccupied vehicle in the grid is replaced by zero. Finally, the interactive tensor of the objective vehicle at moment *t*, with a size of 64*13*3, is obtained. Similarly, for the hidden state $h_{t}^{1}, h_{t}^{2}, \dots ,h_{t}^{n}$ of the interactive information of the surrounding vehicles at any moment in the historical time domain $T_{obs}$ , aforementioned tensor filling operation is repeated to obtain the dynamic interactive tensor $S_{1}, S_{2}, \dots ,S_{T_{obs}}$ in the whole historical time domain. The dynamic interactive tensor is depicted in Fig. 3.

### Extraction of dynamic interactive information

The historical hidden state of the target vehicle extracted by a feature extraction network can be written as:

5 $$\begin{aligned} H_{ego}=(h_{1},h_{2},\dots ,h_{T_{obs}} ) \end{aligned}$$

The hidden state of all neighboring vehicles at moment t can be written as:

6 $$\begin{aligned} H_{t}^{nbr}=(h_{t}^{nbr1},h_{t}^{nbr2},\dots ,h_{t}^{nbru}) \end{aligned}$$

By setting the hidden state $h_{t}$ of the target vehicle at moment *t* as the query, the spatial correlation score with the hidden state $h_{t}^{nbti}$ of the neighboring vehicle at moment *t* , denoted as $l_{t}^{i}$ , can be calculated as follows:

7 $$\begin{aligned} l_{t}^{i}=h_{t}^{T}h_{t}^{nbri} \end{aligned}$$

After normalization of the correlation score, the influence weight of the neighboring vehicle *nbri* on the target vehicle at moment *t* , denoted as $\zeta _{t}^{i}$ , can be written as follows:

8 $$\begin{aligned} \zeta _{t}^{i}=\frac{exp(l_{t}^{i} )}{ {\textstyle \sum _{i=1}^{u}exp(l_{t}^{i} )} } \end{aligned}$$

After attention weighting, the features of all the neighboring vehicles at moment *t* , can be written $H_{t}^{nbr'}$ as:

9 $$\begin{aligned} H_{t}^{nbr'} =\left(h_{t}^{nbr1'},h_{t}^{nbr2'},\dots ,h_{t}^{nbru'}\right), \end{aligned}$$

where $$h_{t}^{nbri'}=\zeta _{t}^{i} h_{t}^{nbri}$$.

**Figure 4:**
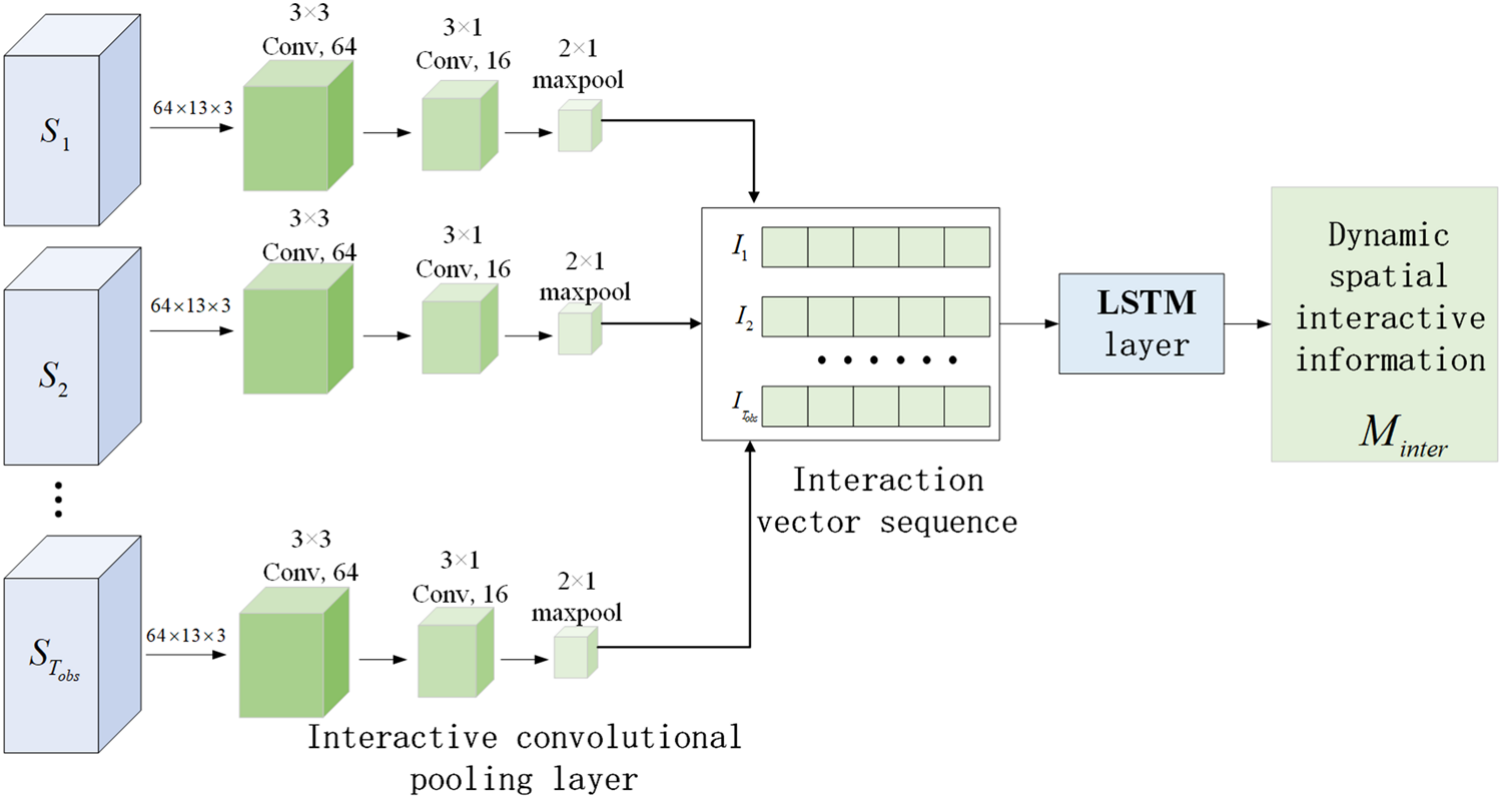
The established dynamic spatial interactive information.

Using the aforementioned grid filling method, the hidden states of all the neighboring vehicles after attention weighting are embedded in the space tensor, and the interactive sensor $S_{1}' ,S_{2}',\dots$ and $S_{T_{obs} }'$ after attention enhancement are obtained. Using CNN, the features of an interactive tensor sequence are extracted in order to obtain additional dynamic interactive information about the vehicle. The time correlation of the interactive vector sequence is learned using an LSTM network in order to preserve the sequential characteristics of the vehicle’s interactive vector. 32 channels comprise the output features of the LSTM coder. Figure 4 displays the overall structure of the dynamic spatial interactive information extraction network.

### Weighting of motion features in historical time domain

**Figure 5:**
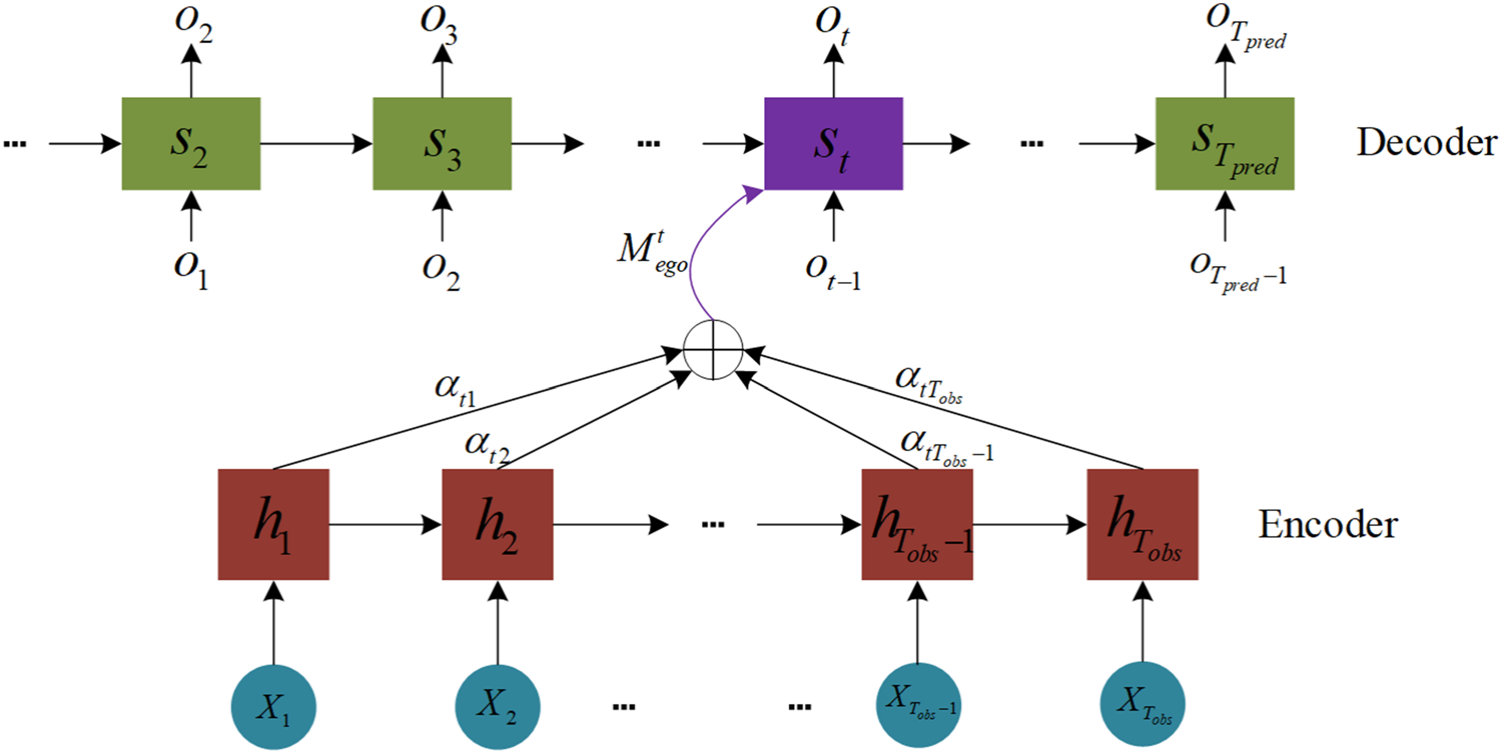
The temporal attention module.

In this study, the temporal attention module is incorporated into the LSTM decoder so that the model can recognize important historical information while avoiding information loss in each decoding step. As shown in Fig. 5, the model estimates the correlation between the information of the encoder and decoder.

During each decoding moment of trajectory prediction, the temporal attention mechanism takes weighted calculation of all hidden states ($h_{1},h_{2},\dots ,h_{T_{obs}}$) in the coder. The degree of correlation between each historical information of the coder at the current decoding moment and the current predicted coordinates is calculated. The required contextual information of the motion features of the target vehicle at current decoding moment, denoted as $M_{ego}^{t}$ , can be reconstructed by the correlation degree:

10 $$\begin{aligned} M_{ego}^{t}=\sum _{i=1}^{T_{obs}}a_{t_{i}}h_{i} \end{aligned}$$

where $\alpha _{t_{i}}$ represents the attention weight and represents the correlation degree between the hidden state $h_{i}$ of the vehicle at moment *i* in the encoder and the state of the encoder at the current moment $s_{t-1}$. $\alpha _{t_{i}}$ is calculated as follows:

11 $$\begin{aligned} \alpha _{t_{i}} =\frac{exp(A(s_{t-1}),h)}{ {\textstyle \sum _{i'}^{T_{obs}}}exp(A(s_{t-1}),h_{i'}) } \end{aligned}$$

where $\alpha _{t_{i}}\in [0,1]$ is the *i*-th component of the attention weight vector $\alpha _{t}$. The attention weight vector $\alpha _{t}$ is calculated by the alignment model $A(\cdot )$:

12 $$\begin{aligned} A(s_{t-1},h_{i})=s_{t-1}^{T}h_{i} \end{aligned}$$

By splicing the resulting dynamic spatial interactive information and the motion information of the target vehicle, the complete contextual vector of the whole trajectory prediction network, denoted as $M_{encoder}$ , can be expressed as follows:

13 $$\begin{aligned} M_{encoder}=[M_{inter};M_{ego}]. \end{aligned}$$

### Trajectory prediction decoder module based on vehicles’ interactions

The vehicles’ interactions modules are composed of fully connected layer with three output nodes. The probability distributions of vehicles’ interactions in each lateral direction are calculated by using the Softmax function:

14 $$\begin{aligned} W=softmax(FC(M_{encoder})), \end{aligned}$$

where $W=(w_{LCL},w_{LCR},w_{LK})$ is the vector consisting of the possibilities of left lane change, right lane change, and straight travelling, respectively. Furthermore, $w_{i}=P(c_{i}|M_{encoder})$ , where $c_{1}$, $c_{2}$, and $c_{3}$ represent the vehicles’ interactions for left lane change, right lane change, and straight traveling, respectively.

In trajectory prediction phase, the probability distribution of the predicted trajectory can be generated based on the recognition of vehicles’ interactions:

15 $$\begin{aligned} P(Y|M_{encoder})=\sum _{i=1}^{3}P_{\theta } (Y_{i}|c_{i},M_{encoder})P_{\theta } (c_{i},M_{encoder}), \end{aligned}$$

where $\theta =[\theta _{T_{obs}+1},\theta _{T_{obs}+2},\dots ,\theta _{T_{obs}+T_{pred}}]$ represents a binary Gaussian distribution parameter comprising the predicted coordinates at each time step in the predicted time domain. It consists of mean, variation, and correlation coefficient of the predicted trajectory at moment $t'$.

By inputting the intention feature vector and the contextual information in the LSTM decoder, a binary Gaussian distribution parameter of the predicted trajectory of the target vehicle at moment $t'$ can be obtained using the fully connected layer:

16 $$\begin{aligned} h_{t'}=LSTM_{decoder}(P(C|M_{encoder}),M_{t'}^{encoder},h_{t'-1}) \end{aligned}$$

17 $$\begin{aligned} \theta _{t'}=FC_{pred}(h_{t};W_{FC_{pred}}), \end{aligned}$$

where $W_{FC_{pred}}$ denotes the learning weight in the fully connected layer for the conversion from the hidden state $h_{t}$ into bivariate Gaussian distribution parameter.

### Design of loss function

Two phases comprise the trajectory prediction model: the prediction of vehicle interactions and the multi-modality trajectory prediction. Self-supervised learning of the entire multi-modality trajectory prediction model can be achieved by designing the loss function precisely.

The first type of loss function is the mean squared error (MSE) between the predicted and actual trajectory of the target vehicle. During calculation, the MSE value can be derived from the predicted trajectory of the target vehicle in real-world driving mode. During the whole prediction process, the MSE loss of the model, denoted as $L_{mse}$, can be written as:

18 $$\begin{aligned} L_{mse}=\frac{1}{T_{pred}}\sum _{t'=T_{obs}+1}^{T_{obs}+T_{pred}}\left((x_{t'}^{*}-x_{t'})^2+(y_{t'}^{*}-y_{t'})^2\right), \end{aligned}$$

where $T_{obs}$ represents the historical observation duration, $T_{pred}$ denotes the predicted duration, ($x_{t'}$, $y_{t'}$) denotes the true trajectory coordinates of the target vehicle, and ($x_{t'}^{*}$, $y_{t'}^{*}$) denotes the predicted trajectory coordinates under real driving mode.

Considering that the value of MSE cannot characterize the degree of uncertainty in the multi-modality trajectory prediction model, the negative log-likelihood (NLL) of the model is used as the loss function:

19 $$\begin{aligned} L_{NLL}=\frac{1}{T_{pred}} \sum _{t'=T_{obs}+1}^{T_{obs}+T_{pred}} log\left(\sum _{i=1}^{3}P_{\theta }(Y_{i}|c_{i},M_{encoder})P_{\theta }(c_{i},M_{encoder}) \right), \end{aligned}$$

where *Y* denotes the output of trajectory prediction phase and $P_{\theta }(Y_{i}|c_{i},M_{encoder})$ denotes the possibility distribution of the predicted trajectory based on multi-modality vehicles’ interactions. The cross-entropy loss between the real vehicles’ interactions and the predicted intention, denoted as $L_{ce}$ , is selected as the third loss function:

20 $$\begin{aligned} L_{ce}=-\sum _{i=1}^{3}\hat{w_{i}}log(w_{i}), \end{aligned}$$

where $\hat{w_{i}}$ represents the real intention of the target vehicle and $w_{i}$ denotes the prediction probability of vehicles’ interactions classifications of the target vehicle.

For accelerating the convergence of the prediction model, the cross-entropy loss of vehicles interactions ($L_{ce}$) and the MSE value of the predicted trajectory ($L_{mse}$) are selected as the loss function during the pre-training phase $L_{pred1}$:

21 $$\begin{aligned} L_{pred1}=\alpha _{1}L_{ce}+\beta _{1}L_{mse} \end{aligned}$$

To reduce the uncertainty in the generated trajectory using the multi-modality trajectory prediction model, the cross entropy loss of vehicles’ interactions ($L_{ce}$) and the NLL value ($L_{mse}$) are selected as the loss function during formal training of the model ($L_{pred2}$):

22 $$\begin{aligned} L_{pred2}=\alpha _{2}L_{ce}+\beta _{2}L_{NLL}. \end{aligned}$$

## Data processing

### Introduction of the data set and feature selection

**Figure 6:**
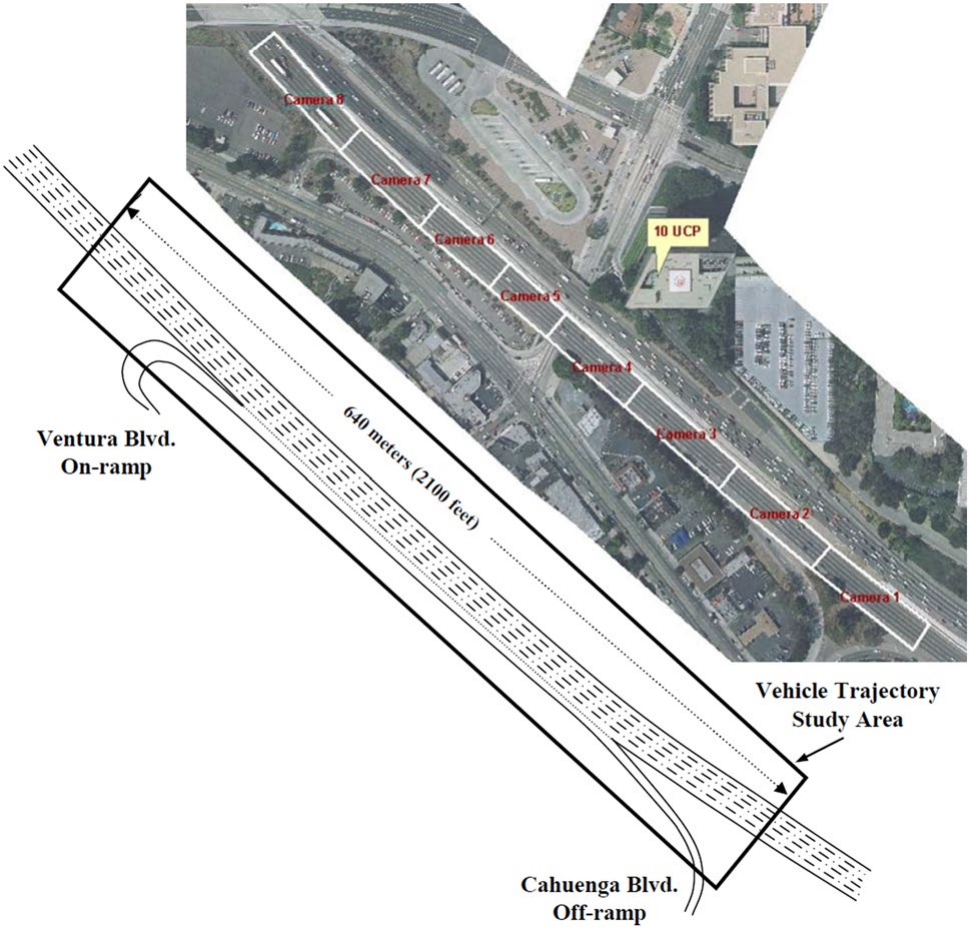
A road segment sample from US-101 dataset.

**Figure 7:**
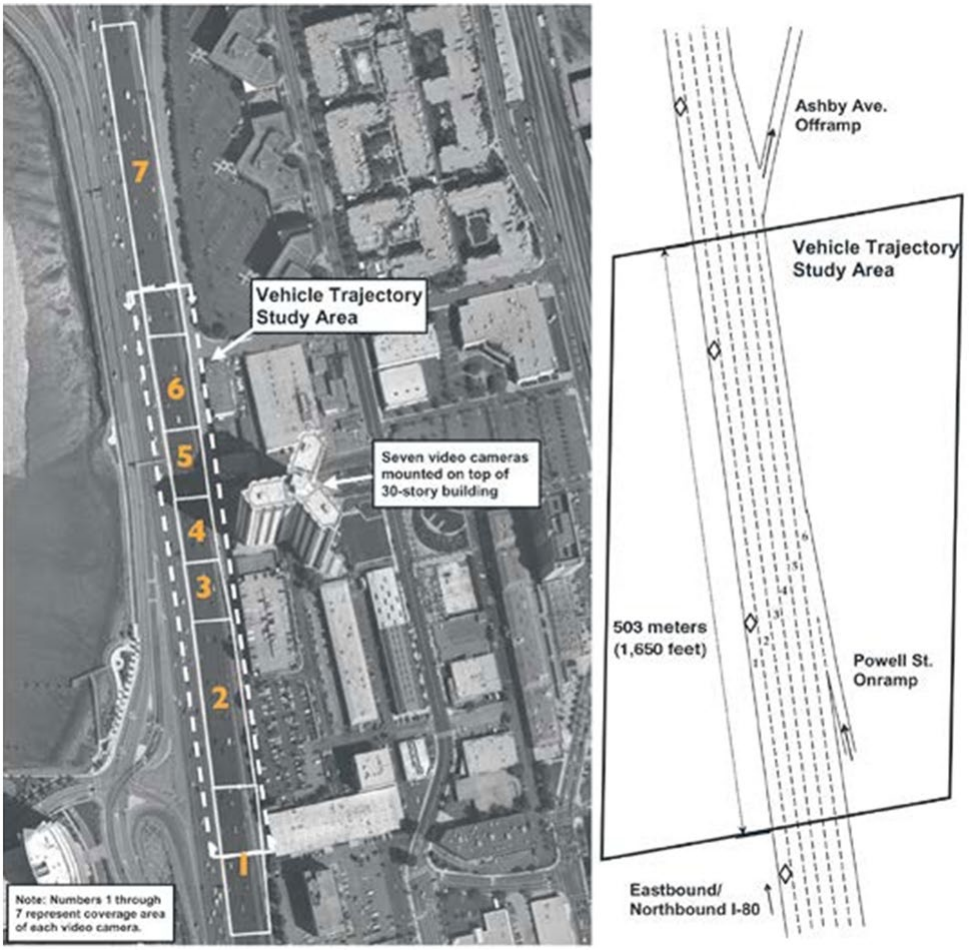
A road segment sample from I-80 dataset.

For the experiments, this study selects vehicle travel data from two high-speed road datasets (US-101 and I-80) in the NGSIM^25^ project funded by the Federal Highway Administration of the United States. Figures 6 and 7 depict the road sections examined in this study. The selected datasets (US-101 and I-80) contain information on vehicles on high-speed roads during different periods (light, moderate, and severe congestion). The combined duration of various time periods is 45 min. At a sampling rate of 10 Hz, the multi-field information primarily related to vehicle lane change and following behaviors, including serial number, horizontal and longitudinal positions, velocity, acceleration, vehicle type, and lane number, is collected.

In addition to the vehicle’s historical trajectory information, instantaneous velocity, acceleration, and lane ID information of the main vehicle from the historical time domain are added in the selection of input features for enhancing the feature expression of vehicle trajectory.

The overall input features of the model can be written as follows:

23 $$\begin{aligned} X=(X_{1},X_{2},\dots ,X_{T_{obs}}) \end{aligned}$$

where $X_{t}$ represents the vehicle’s historical features and Tobs denotes the total observation time steps. The input features at moment *T* can be written as follows:

24 $$\begin{aligned} X_{t}=(x,y,v,a,l), \end{aligned}$$

where *x* and *y* represent the vehicle’s local x-axis and y-axis coordinates, *v* denotes the vehicle’s instantaneous velocity, *a* denotes the vehicle’s instantaneous acceleration, and *l* represents the lane number of the vehicle.

### Data pre-processing

**Figure 8:**
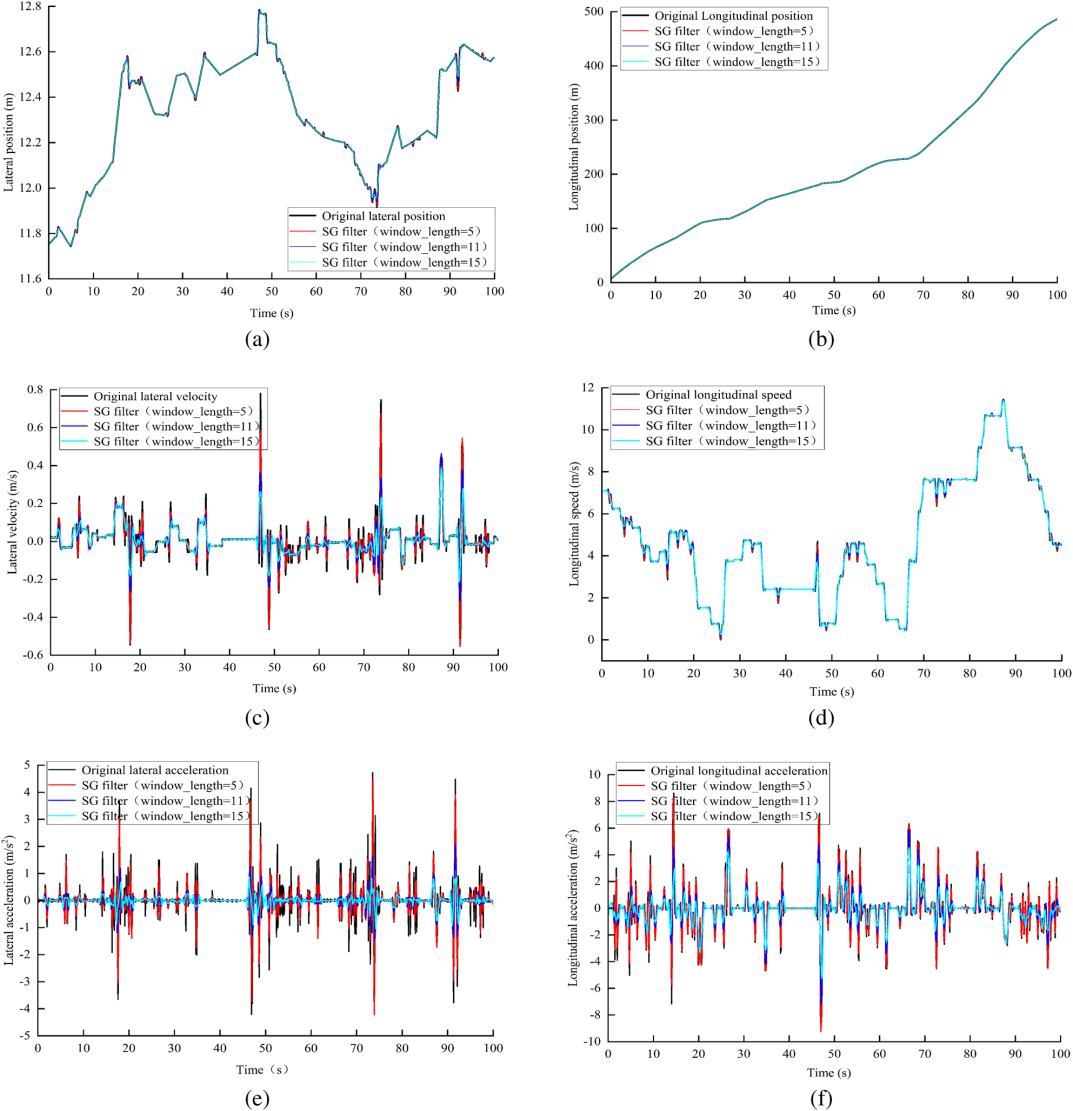
Smoothing processing on NGSIM data. (**a**) Smoothing on lateral position data; (**b**) Smoothing on longitudinal position data; (**c**) Smoothing on lateral velocity data; (**d**) Smoothing on longitudinal velocity data; (**e**) Smoothing on lateral acceleration data; (**f**) Smoothing on longitudinal acceleration data.

To address the noise problem in the NGSIM original data, this study used the Savitsky-Golay filtering algorithm to denoise the vehicle’s horizontal and longitudinal positions, horizontal and longitudinal velocities, and horizontal and longitudinal accelerations. The corresponding results are shown in Fig. 8. Finally, the optimal window lengths for denoising the horizontal and longitudinal positions, horizontal and longitudinal velocities, and horizontal and longitudinal accelerations are 11, 15, and 15, respectively. The implementation procedures are described in detail as follows:

25 $$\begin{aligned} x^{*}(t_{i})=\frac{1}{2a+1}\sum _{j=-a}^{a}w_{j}x(t_{i+j}), \end{aligned}$$

where $x^{*}(t_{i})$ denotes the data after denoising, $x(_{i+j})$ denotes the original data, $2a+1$ is the length of the siding window, and $w_{j}$ denotes the weight factor.

In order to avoid the vanishing gradient of the LSTM network during the processing of contextual information in long-term domain data, preserve the important trajectory features, and reduce the time span, we downsampled each feature segment by a factor of 2 prior to feeding them into the LSTMs. The downsampling frequency is 5 Hz, which corresponds to a time interval of 0.2 s. After downsampling, the surrounding vehicles’ interactive information is added by filling the interactive tensor. For training, validating, and testing the model, the dataset is divided into three sets with a 7:1:2 ratio. The window with a size of 8 s and a time interval of 0.2 s is slid on the dataset iteratively so as to obtain the training samples. The first 16 samples of trajectory data serve as the historical input for the prediction model, while the last 25 samples serve as the ground truth for calculating the deviation between the predicted and actual trajectory.

## Experimental validation and analysis

### Design of experimental parameters

The STA-LSTM prediction model is compiled with Python 3.6, and established based on the deep learning framework Pytorch 1.6.0. The experimental platform is constructed with NVIDIA GeForce RTX 2070 super GPU and Intel Core i7-10700K CPU.

The super-parameters in STA-LSTM trajectory prediction model during the experiment are set as follows:

1. The word embedding dimensionality in the coding layer is set to 32.
2. LSTM encoder and decoder adopt 64-dimensional and 128-dimensional states, respectively.
3. The batch size is set to 128.
4. ReLU activation function is used in the word embedding layer and convolutional pooling layer.
5. The initial learning rate is set to 0.001, and Adam optimizer is used for optimizing the weights of the network.

### Analysis of the training process

**Figure 9:**
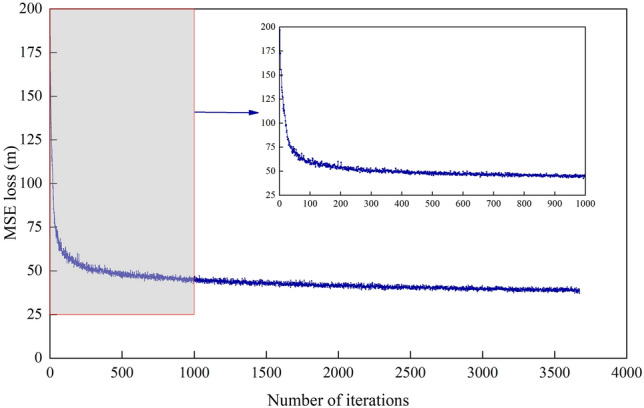
The variation curve of the MSE loss with the iteration number.

**Figure 10:**
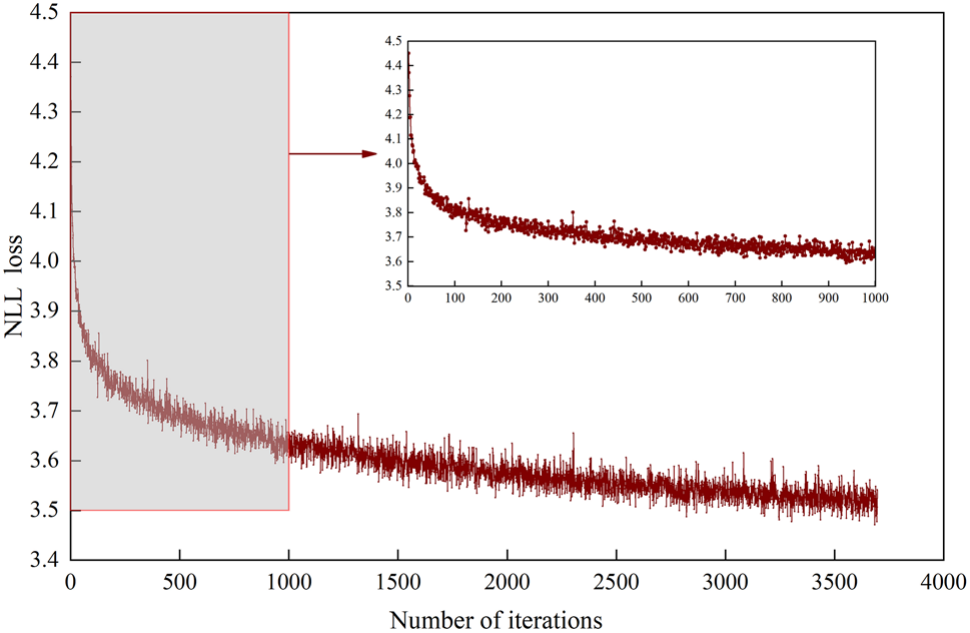
The variation curve of the NLL value with the iteration number.

Figures 9 and 10 show the loss curves of $L_{mse}$ and $L_{NLL}$ during the training process of the STA-LSTM model. At the beginning of pre-training, the MSE loss of the model is 193.58 m. After 100 iterations, the MSE loss gradually converges to a range of [38, 40]. During normal training, the prediction model finally converges to [3.50, 3.60]. After two training phases, the loss value gradually converges with an increase in the iteration number, and no over-fitting is observed.

### Analysis of the comparison of the predicted errors

In this study, root mean square error (RMSE) and NLL of the predicted trajectory are selected as the model’s performance evaluation indexes, which are calculated as follows:

26 $$\begin{aligned} L_{rmse}=\sqrt{\frac{1}{N\times T_{pred}}\sum _{i=1}^{N}\sum _{t'=T_{obs}+1}^{T_{obs}+T_{pred}}(x_{t'}^{*}-x_{t'})^2+(y_{t'}^{*}-y_{t'})^2) } \end{aligned}$$

27 $$\begin{aligned} NLL=\frac{1}{N\times T_{pred}}\sum _{i=1}^{N}\sum _{t'=T_{obs}+1}^{T_{obs}+T_{pred}}log\left(\sum _{i=1}^{3}P_{\theta }(Y_{i}|c_{i},M_{encoder})P(c_{i},M_{encoder}) \right). \end{aligned}$$

For the purpose of validating the overall performance of the proposed multi-modality trajectory prediction results, the prediction performances on the test set obtained using the proposed STA-LSTM model and the current mainstream trajectory prediction models are compared based on the aforementioned evaluation metrics, during which the historical time-domain information for a duration of 3 s was input. In this study, the following baseline models are utilized.

Vanilla LSTM (V-LSTM): features of the vehicle’s historical trajectory are extracted using an LSTM encoder, and a single-modal predicted trajectory is generated using an LSTM decoder.

Social LSTM (S-LSTM)^18^: the traditional pedestrian trajectory prediction model taking into account social factors. Interactive information is transplanted into the trajectory of a vehicle, a social pool is created based on the vehicle’s relative position, and social features are extracted using a fully connected layer.

Maneuver-LSTM (M-LSTM)^26^: a multi-modality prediction model based on encoder-decoder architecture is used to account for the motion states of the surrounding vehicles.

Convolutional social LSTM (CS)^27^: the interaction feature extraction network of S-LSTM is enhanced, and the convolutional social pool is used to extract the interaction information between adjacent vehicles.

Table 1 contains the RMSE and NLL values derived from the NGSIM dataset using various prediction models. The errors are calculated at an interval of 1 s. To be specific, for the multi-modality trajectory prediction model, the predicted trajectories under true driving conditions are selected for the calculation of RMSE.

**Table 1:** A comparison of RMSE and NLL values obtained using different prediction models.

Evaluation index	Prediction time	V-LSTM	S-LSTM	M-LSTM	CS-LSTM	STA-LSTM
RMSE(m)	1s	0.68	0.65	0.58	0.62	0.46
	2s	1.65	1.31	1.26	1.29	1.15
	3s	2.91	2.16	2.12	2.09	1.89
	4s	4.46	3.35	3.24	3.1	2.84
	5s	6.27	4.55	4.66	4.37	4.05
NLL	1s	1.17	1.01	0.56	0.58	0.49
	2s	2.85	2.49	2.13	2.14	2.06
	3s	3.8	3.36	3.12	3.03	2.92
	4s	4.48	4.01	3.71	3.68	3.54
	5s	4.99	4.54	4.39	4.23	4.11

As shown in Table 1, various prediction models differ slightly in terms of the RMSE value of short-term prediction. However, as the prediction time increases, the V-LSTM model, without consideration of inter-vehicle interactions, performs the worst. This is due to the fact that short-term predictions heavily rely on the vehicle’s most recent traveling state, and that a model based solely on the vehicle’s historical trajectory cannot estimate the high non-linearity of long-term predictions. Among the models considering vehicle interaction, the classical pedestrian trajectory prediction model (S-LSTM) cannot effectively address the vehicle trajectory prediction problem under high-speed scenarios. Using multi-modality prediction models that take vehicle interactions into account, CS-LSTM’s short-term prediction performance is inferior to that of M-LSTM. However, as the prediction time increases, the RMSE and NLL values after 5 s of prediction are 0.29 m and 0.16 m less than the values obtained using M-LSTM. This indicates that the convolutional network effectively retains the benefits of spatial data when modeling vehicle interaction. Multi-modality prediction model outperforms V-LSTM and S-LSTM models in terms of NLL value, indicating that the established multi-modality prediction model based on the recognition of vehicles’ interactions can make deterministic predictions on the distribution of future trajectories.

In contrast, the STA-LSTM model incorporates a spatial-temporal attention mechanism in addition to the dynamic interaction between vehicles. In long-term prediction, the model pays more attention to the interactive features that have a significant effect on the target vehicle, whereas in short-term prediction, it considers the most relevant historical information. The STA-LSTM model performed the best in terms of prediction among all models. Using the STA-LSTM model, the mean RMSE and NLL values at various prediction times are reduced to 2.078 m and 2.624 m, respectively.

### Ablation experiment

Ablation experiments are performed to evaluate the degree of contribution of each module in STA-LSTM to the prediction performance, with the NLL value serving as the evaluation index. The experimental results are presented in Table 2.

In the first ablation experiment, only the vehicle’s position information is adopted as the model input. Table 2 demonstrates that adding the vehicle’s motion information and road constraints can significantly improve the model’s performance. When both spatial and temporal attention modules are removed, the established model performs poorly for long-term predictions, with NLL values at 5 s prediction time increasing by 0.11 and 0.18, respectively. This suggests that the addition of an attention mechanism and multi-channel convolution to the modeling of dynamic interaction between vehicles can effectively improve prediction accuracy. When the recognition module of vehicle interactions is peeled, the single-modal STA-LSTM prediction model performed the worst in terms of NLL value, with a 0.352% increase in the mean NLL value compared with the STA-LSTM model. This suggests that the multi-modality prediction distribution based on the recognition of vehicle interactions is most aligned with the actual driving trajectory, indicating that multi-modality is a feature of the trajectory prediction task.

**Table 2:** The results of ablation experiments.

Evaluation index	Prediction time	Only input (x,y)	Without	Without	Without	STA-LSTM
			S-At	T-At	Maneuver	
NLL	1s	0.56	0.52	0.53	0.85	0.49
	2s	2.1	2.09	2.12	2.37	2.06
	3s	3.11	2.99	3.04	3.28	2.92
	4s	3.59	3.61	3.65	3.89	3.54
	5s	4.16	4.22	4.29	4.49	4.11

### Analysis of multi-modality prediction performance

**Figure 11:**
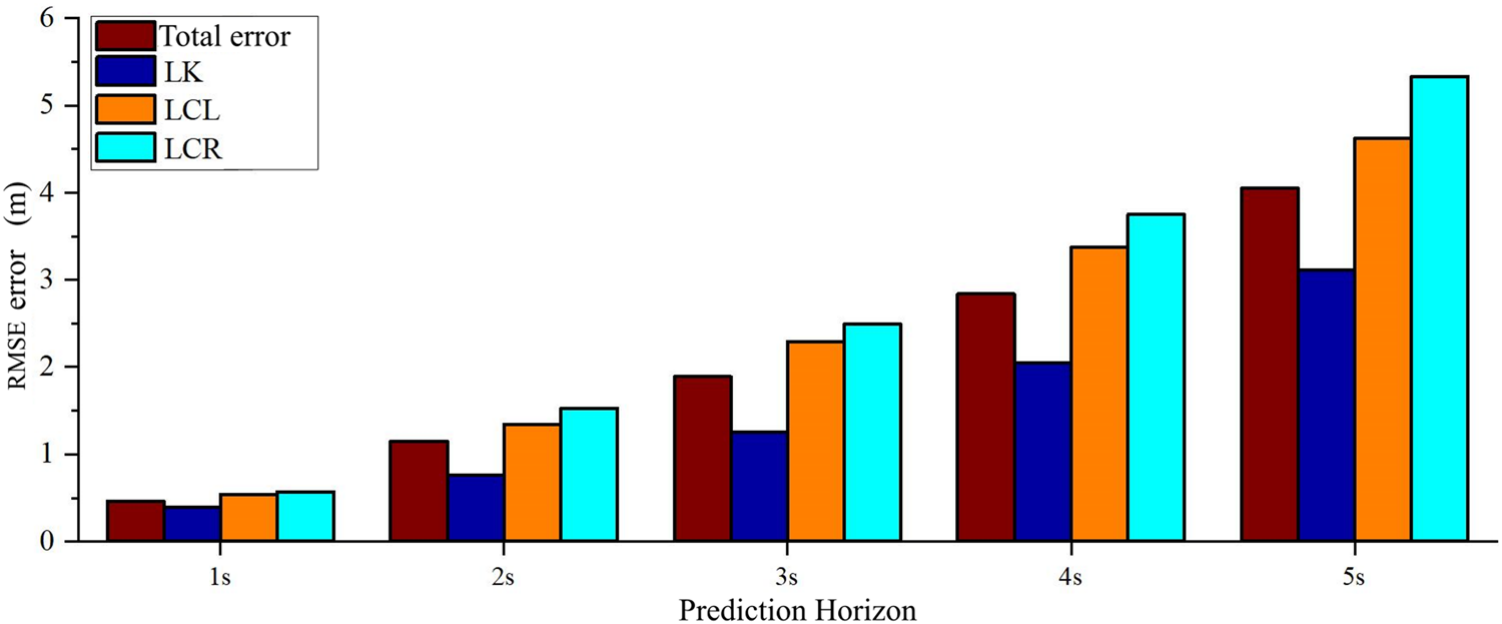
A comparison of the RMSE values of the predicted trajectories under different vehicles interactions.

According to the results of the ablation experiment described in Section 3.4, the NLL value of the model increased significantly after the recognition module of vehicle interactions was removed. In this section, the effect of vehicle interactions on the prediction performance of the model is examined, and the performance of multi-modality and single-modality models is compared. The horizontal vehicle interactions in the dataset are redivided into lane keeping (LK), lane change towards the left (LCL), and lane change towards the right (LCR) samples, respectively. As shown in Fig. 11, using the STA-LSTM model, the MSE values of the predicted trajectories of the above three types of vehicles are calculated within 5 s.

It can be observed that the STA-LSTM model has the lowest MSE and highest prediction precision for LK vehicles. Using the STA-LSTM model, the MSE values of predicted LCL and LCR vehicle trajectories are obviously greater than the value for the entire dataset. Considering STA-LSTM based on three vehicles interactions, the recognition accuracies of LK, LCL, and LCR are 93.27%, 88.65%, and 86.37%, respectively. It can be observed that the precision of the model’s trajectory prediction is positively correlated with the accuracy of the driving intention recognition module. The precision of the model’s trajectory prediction can be improved by incorporating the accuracy of the model’s intention recognition.

**Figure 12:**
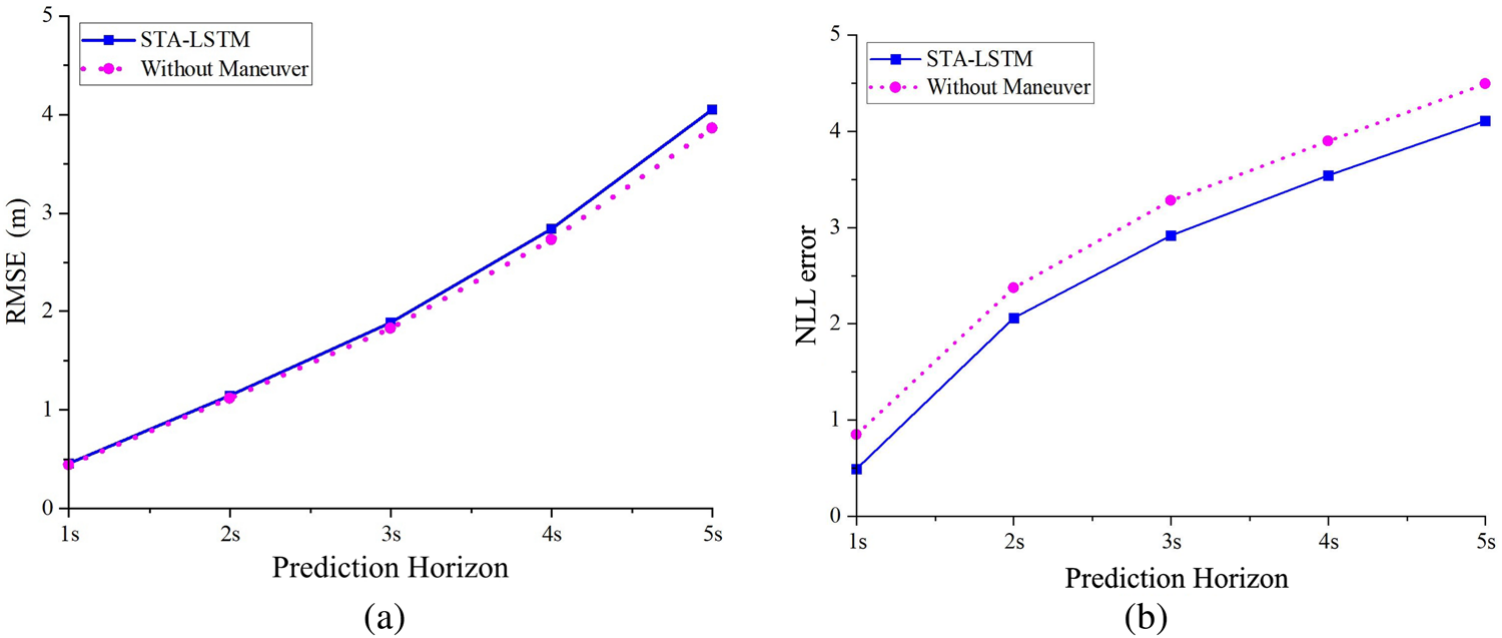
A comparison between single-modal and multi-modality prediction errors. (**a**) Comparison of the MSE; (**b**) Comparison of the NLL.

This study selects RMSE and NLL as the evaluation indices for both single-modal and multi-modal prediction modes in order to further investigate the effect of the recognition module of vehicle interactions on the trajectory prediction performance of the model. The prediction errors are shown in Fig. 12.

The single-modal prediction model, after removing the recognition module of vehicle interactions, outperformed the original STA-LSTM model in terms of RSME, whereas the multi-modal STA-LSTM trajectory prediction model had a much lower NLL value than the single-modal model. The primary causes are outlined below. The RMSE index measures the mean error between predicted trajectory points and actual values and is more appropriate for evaluating models that produce mean modes. The multi-modality prediction model aims to generate the probability distribution of the predicted trajectories, with certain directivity. The mean error cannot be used to quantify the probability distribution of multiple modes. The single-modal model outputs the mean predicted trajectories for lane-changing vehicles, failing to characterize the vehicle’s future behavior motive. Clearly, this is inconsistent with actual driving scenarios. Overall, the complete multi-modality prediction model STA-LSTM reflects uncertainty in the future travel trajectory of a vehicle.

### Visualized analysis of the predicted results

#### Visualized analysis of the predicted trajectories

**Figure 13:**
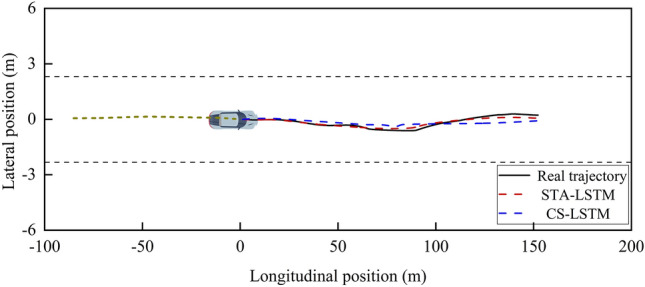
Visualization of the predicted trajectories under a single-vehicle straight-traveling scenario.

**Figure 14:**
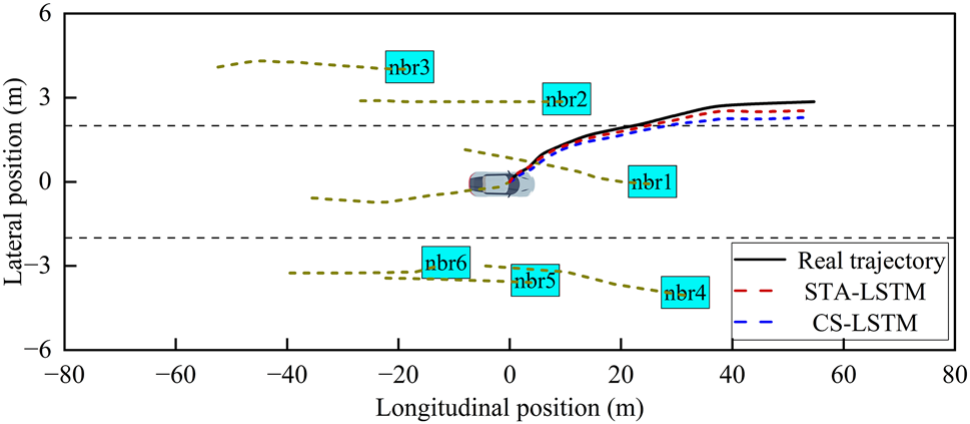
Visualization of the predicted trajectories under a multi-vehicle interaction scenario.

This study selects single-vehicle straight-traveling and multi-vehicle lane-changing scenarios from the test set for the visualization of predicted trajectories in order to qualitatively analyze the advantage of the proposed STA-LSTM model in comparison to the baseline CS-LSTM model more clearly. The results are depicted in Figs. 13 and 14, where the historical trajectories of the target vehicle and the surrounding vehicles are indicated by yellow dashed lines, the true trajectory of the target vehicle is indicated by a solid black line, and the trajectories predicted by STA-LSTM and CS-LSTM are indicated by red and blue dashed lines, respectively.

As shown in Fig. 13, the straight vehicle travels quickly in a no-interaction scenario, and the predicted trajectory of the vehicle using STA-LSTM is closer to the actual trajectory than the trajectory obtained using CS-LSTM. As shown in Fig. 14, under conditions of moderate traffic flow, the interaction between the target vehicle and its neighboring vehicles can have a significant impact. The intention of left lane change came out and the driver changed the lane. The proposed STA-LSTM model captures the interaction between vehicles accurately. Using STA-LSTM, the predicted trajectories in the entire prediction time domain slightly deviated from the actual traveling trajectories. Particularly, predicted trajectories in the early stages of prediction are nearly identical to actual trajectories. Conclusively, due to the introduction of the attention mechanism, the proposed model captures the interactive information with high correlation degree with future predicted trajectories and the vehicle’s historical information, thereby enhancing the model’s understanding of dynamic spatial interaction among vehicles.

#### Analysis of the weight of spatial attention

**Figure 15:**
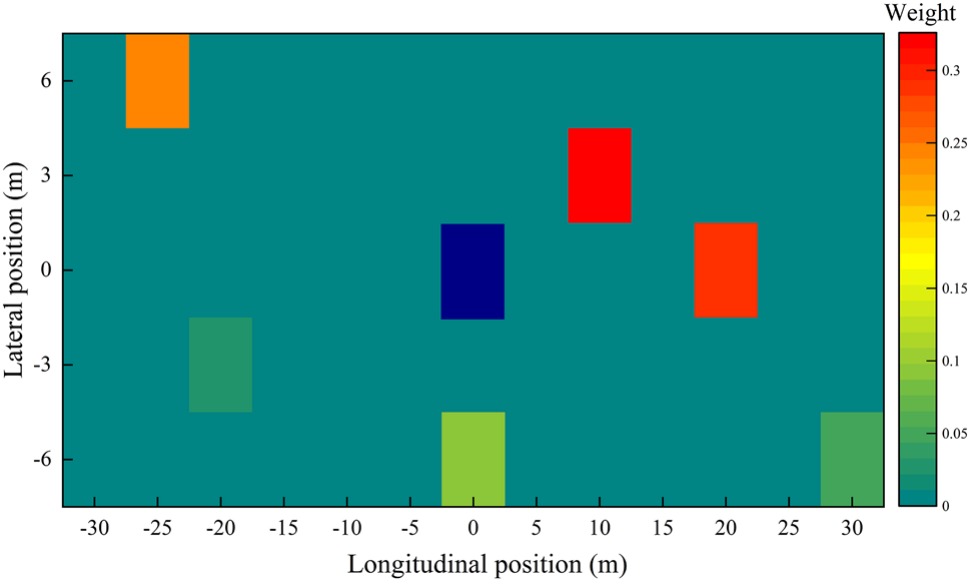
The spatial attention weights of the neighboring vehicles.

In order to explore which neighboring vehicles aroused more attention by using the spatial attention mechanism in the STA-LSTM model during the prediction process, the spatial attention heat map under the LCL scenario in Fig. 14 is plotted for visualization analysis. Figure 15 depicts a heat map in which the target vehicle is denoted by a blue rectangle and the associated region of the vehicle is denoted by a cyan rectangle. The attention weights of the surrounding neighboring vehicles are marked by other colors. A darker color indicates a greater influence on the target vehicle.

As shown in Fig. 15, the model’s attention is primarily focused on the vehicle in front of the target vehicle and the vehicle in the target lane, similar to the driver’s driving behavior. Moreover, although the neighboring vehicle, nbr5 in the right lane, is closer to the target vehicle than other vehicles, the model does not assign it a higher degree of attention. This suggests that the closest neighboring vehicle does not always exert the greatest effect on the travel trajectory of the target vehicle.

#### Analysis of temporal attention weight

**Figure 16:**
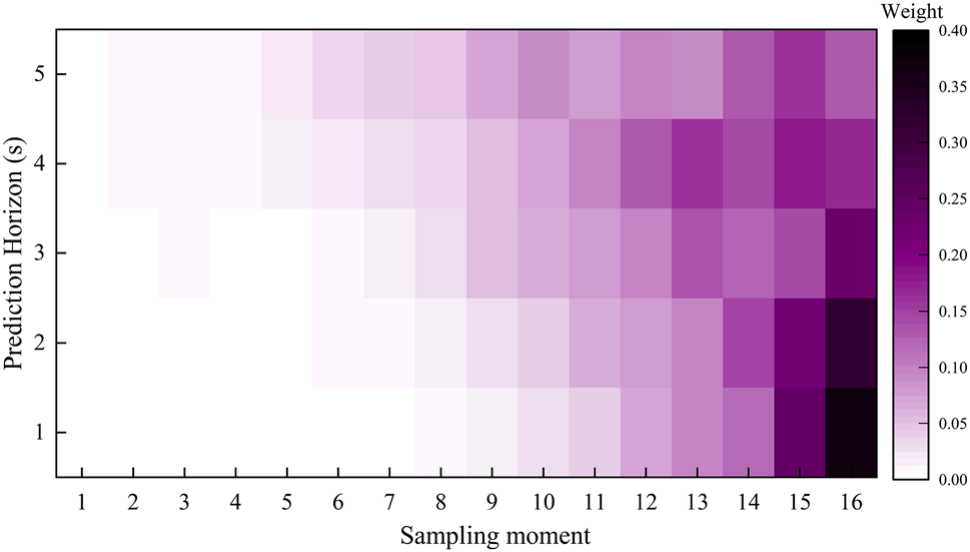
The attention weights at different historical moments.

The past state of the vehicle is highly correlated with its future travel path. In addition, the correlation varies dynamically at various prediction and historical times. This study introduced the attention mechanism from time perspective for modeling the correlation between history and future states of trajectory. Figure 16 shows the heat map of the attention weights of the above lane-changing vehicles at various times in the past. Throughout the experiment, the correlation weight between historical data and the predicted trajectory is calculated at an interval of 1 s.

Figure 16 displays the heat maps of the weights at different past moments after the prediction of 1 s, 2 s, 3 s, 4 s, and 5 s, respectively. Apparently, the attention weight distribution of historical data varies in real-time at various prediction moments. This indicates that the temporal attention mechanism module can provide a real-time response to alter the vehicle’s traveling state, comprehend the hidden states at all past time steps in the vehicle’s information encoder, and effectively capture the historical information most relevant to the predicted trajectory, thereby facilitating future trajectory prediction. In addition, the heat map of the attention weight reveals that the weight at the past moment close to the current moment is relatively high, indicating that the future traveling trajectory of the vehicle is highly dependent on the state history at the most recent time moment. As the prediction time increases, the correlation between the predicted and historical trajectories is weakened, and the weight of the information at the later past moment drops gradually. It can be concluded that the model can adaptively distribute the weights of the vehicle’s historical information and delete unimportant historical information.

## Conclusion

In the context of connected vehicle environments, complex multi-vehicle interaction scenarios present challenges where the relative motion and spatial positioning of vehicles are inaccurately characterized in the temporal and spatial dimensions. Additionally, the dynamic interplay between vehicles complicates the use of simplistic linear models for accurate trajectory prediction. To address this issue, this study proposed a multi-modality trajectory prediction model STA-LSTM based on spatial-temporal attention and modeled the interaction between vehicles with space grid occupancy. Moreover, the attention module is embedded in the LSTM decoder from the perspective of time so that the model can identify significant hidden historical features in each trajectory during the decoding process. According to the experimental results on the NGSIM dataset, the proposed prediction model can effectively derive dynamic interaction between vehicles, with favorable recognition performance of vehicles’ interactions and high trajectory prediction precision, thereby enhancing the intelligent and connected vehicle’s comprehension and cognitive capabilities of the current traffic situation.

The proposed trajectory prediction model is developed based on high-speed travel scenarios, excluding urban intersections and unstructured road scenarios. In future research, we will focus more on additional traffic scenarios in order to improve the generalization performance of the model.

## Data Availability

The data that support the findings of this study are openly available in [Traffic Analysis Tools: Next Generation Simulation-FHWA Operations] at [https://ops.fhwa.dot.gov/trafficanalysistools/ngsim.htm].
